# Investigation on the Temperature Distribution Uniformity of an Extrusion-Based 3D Print Head and Its Temperature Control Strategy

**DOI:** 10.3390/pharmaceutics14102108

**Published:** 2022-10-01

**Authors:** Qiang Gao, Kaicheng Yu, Fangzheng Chen, Lihua Lu, Peng Zhang

**Affiliations:** 1 School of Mechatronics Engineering, Harbin Institute of Technology, Harbin 150001, China; 2 Chongqing Research Institute of HIT, Chongqing 401120, China; 3 School of Astronautics, Harbin Institute of Technology, Harbin 150001, China

**Keywords:** extrusion-based 3D printing, temperature distribution, rheological properties, thermoplastic polymer

## Abstract

Extrusion-based 3D printing for thermoplastic polymers manifests potential for the fabrication of biocompatible and biodegradable scaffolds. However, the uncontrollable shape of printed filaments usually negatively impacts on the printing processes. Non-uniform temperature in the print head is a primary cause of inaccuracy in the diameter of filaments formed during the process of extruding thermoplastic polymers. Therefore, the temperature distribution inside the print head must be controlled accurately. This study developed a novel print head configuration with two groups of controllable heat sources for extrusion-based printing of thermoplastic polymers. Subsequently, a numerical thermal analysis based on the finite element method (FEM) was conducted to investigate the temperature field in the print head during the heating process. Moreover, a temperature control strategy is proposed under which the temperature distribution of the print head can be regulated. The temperature uniformity can be improved with the proposed temperature control strategy. Lastly, groups of printing trials were implemented, and the printed filaments showed excellent uniformity of diameter when temperature distribution uniformity was controlled in the print head.

## 1. Introduction

As an emerging manufacturing technique for physiologically relevant models of tissues and organs, three-dimensional (3D) printing is widely acknowledged in the fields of tissue engineering and regenerative medicine [1,2]. Complex geometric structures can be fabricated with various biocompatible materials via a layer-by-layer printing process [3,4].

To date, 3D printing technologies based on photopolymerization [5,6,7], inkjet [8,9,10], and extrusion [11,12] have been three of the commonly employed methods in biological engineering. The extrusion-based 3D printing process is relatively economical and convenient to operate [13]. Consequently, it has been developed into the most frequently employed method among the aforementioned three printing techniques. Extrusion-based 3D printing devices can be customized to adjust the rheological properties of printing materials, which indicates that they are compatible with a wide variety of bio-ink materials. In the printing process, the bio-ink materials are typically loaded into a cartridge, and are then extruded into continuous filaments under the influence of mechanical force or pneumatic pressure. These filaments are then deposited onto a two-axis linear stage with a predefined trajectory, and a complex 3D geometric structure can ultimately be fabricated.

A wealth of studies have been conducted in biological engineering with extrusion-based 3D printing technology. Merceron et al. [14] manufactured a muscle–tendon unit construct using extrusion-based 3D printing. NIH/3T3 cell-laden hydrogel-based bio-ink and thermoplastic polymers were adopted to fabricate the complex construct via two separate print heads. The printed structure demonstrated dissimilar tensile properties at the muscle and tendon phases. Lee et al. [15] constructed a tri-leaflet heart valve at an adult human scale via extrusion-based 3D printing. Alginate and collagen were adopted to print the valve models. The mechanical function and robustness of the collagen constructs were experimentally revealed in a flow system. Kang et al. [16] fabricated a 3D architecture of mandible bone fragments by extrusion-based 3D printing. Blends of PCL and tricalcium phosphate, Pluronic F127, and cell-laden hydrogel were employed to construct the complex structure. Human amniotic fluid-derived stem cells were cultured on the fragment and the potential of prepared structure in mandible bone reconstruction was revealed. Miller et al. [17] developed a monolithic tissue construct with patterned vascular structures via extrusion-based 3D printing. Carbohydrate glass was printed and subsequently dissolved to create open channels in the architecture of fibrin. The developed tissue construct manifested the capability to maintain the biological function of primary rat hepatocytes. Hinton et al. [18] fabricated 3D architectures of the human femur and right coronary arterial tree via extrusion-based 3D printing. Researchers utilized alginate to print the complex structures and embed them in the gelatin slurry support bath. The models demonstrated similar shapes with the 3D computed tomography imaging.

Generally, the bio-ink materials adopted in extrusion-based 3D printing techniques are biocompatible thermoplastic polymers. These polymers reveal splendid mechanical and degradation properties at body temperature, which are appropriate for implantable scaffolds in various cases, such as bone repair and wound healing [19]. However, the extruding process of thermoplastic polymers is usually uncontrollable because their rheological properties are susceptible to temperature variation [20]. Plenty of materials, such as poly(ɛ-caprolactone) (PCL), strontium-containing hydroxyapatite (SrHA), silver nanoparticles (AgNps), polydioxanone (PDO), and polylactide-co-caprolactone (PLCL) cannot be printed until they are heated to suitable temperatures. Hence, the temperature of the print head should be precisely controlled as a crucial process parameter. Liu et al. [21] mixed PCL and SrHA into blends to fabricate composite scaffolds. PCL/SrHA blends with a formulation of 50:50 were heated to 200 °C to ensure a continuous extrusion process. Rat bone marrow-derived mesenchymal stem cells were planted on prepared scaffolds and the capability to facilitate the cell proliferation of composite scaffolds was revealed. Radhakrishnan et al. [22] prepared a scaffold that exhibited interconnected porous structures via extrusion-based 3D printing. The composites of AgNps and PCL were prepared at 90 °C and then printed at 120 °C. The potential of the fabricated scaffolds for tissue engineering was demonstrated by analyses of enzymatic degradation and antibacterial activity. Lee et al. [23] fabricated a vascular graft with melts of PCL and PDO. They adopted an extrusion-based 3D printing device to deposit the PCL filaments onto a prepared nanofibrous PCL/PDO scaffold at 90 °C. The vascular graft was implanted into pigs for four weeks and the potential of PCL/PDO vascular grafts in vascular reconstruction was manifested. Chang et al. [24] employed an extrusion-based 3D printer to develop a skin model for tissue engineering. The PLCL melts were printed at 150 °C to fabricate a 3D scaffold. Minimum functional units of rat tail skin were subsequently loaded into the printed PLCL scaffolds to prepare a functional skin model which was capable of expediting the wound healing process.

Although the there is broad consensus on the crucial influence of temperature on the process of 3D printing thermoplastic polymers, the uniformity of temperature distribution inside the print heads is seldom investigated. In extrusion-based 3D printing, bio-ink materials are loaded in a cartridge and heated by the print head. The temperature distribution inside the print head might vary spatially depending on the location of the installed heat sources, thermal conduction of mechanical structures, and convective heat transfer with the surrounding air [25]. Since the rheological properties of thermoplastic polymers are sensitive to temperature variation, the spatial temperature distribution of the print head could eventually determine the fluidity of the materials at different locations [26]. If the temperature gradient of the print head is too great, it can lead to a dramatic change in the rheological properties of bio-ink materials in different locations. This means the shape of the filament will be non-uniform, thus decreasing the accuracy of the diameter of the printed filament and consequently the accuracy in the shape of the printed structure, or even lead to a collapse in the depositing process. Furthermore, as the 3D printing of complex architecture usually requires a continuous and time-consuming process, this problem would result in a waste of bio-ink materials and time. Hence, the spatial uniformity of temperature distribution in the print head is critical to control in extrusion-based 3D printing.

In this paper, the impact of the spatial uniformity of temperature distribution within the print head on the accuracy of the diameter of printed filaments was investigated. A novel configuration of print head with controllable heat sources is proposed for regulating the temperature distribution of the print head. An FEM-based simulation model was employed to numerically analyze the temperature distribution in the print head. Furthermore, a temperature control strategy was established to improve the uniformity of spatial temperature distribution in the designed print head, and this temperature control strategy was adopted to conduct printing trials. It was found that filament diameter accuracy can be improved by the proposed control strategy for temperature distribution.

## 2. A Novel Configuration of Extrusion-Based 3D Print Head

**Figure 1:**
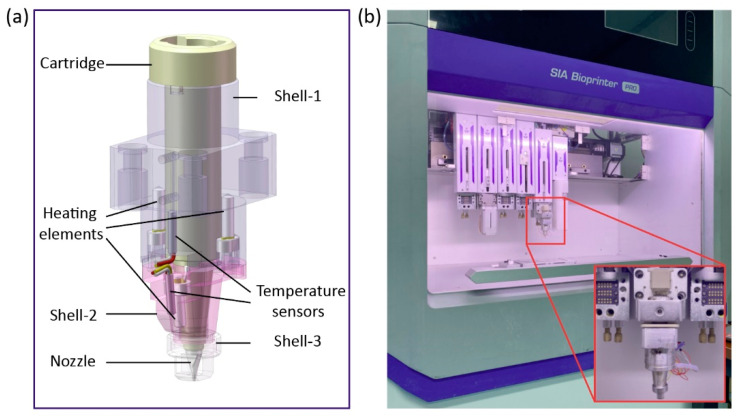
Configuration of the 3D printing device. (**a**) Configuration of the proposed print head; (**b**) A self-developed high-precision 3D bioprinter.

Existing configurations of extrusion-based 3D print heads usually contain only a single heat source. Since the dimensions of the print head are long in the axial direction relative to the radial direction, a single heat source is incapable of providing a uniform temperature distribution to the entire print head. The temperature at locations further away from the heat source will inevitably be different from that at locations close to the temperature source, due to the convective heat transfer between the print head and surrounding air. To overcome this problem, a novel print head configuration of is proposed in this paper which can improve the uniformity of temperature distribution in the bio-ink materials, as shown in Figure 1a.

The proposed print head configuration consists of a cartridge, three shells, three heating elements, two temperature sensors, and a nozzle. The cartridge is made of aluminum alloy, which is utilized for accommodating and heating the bio-ink materials. Two aluminum alloy shells (shell-1 and shell-2) are adopted to cover the cartridge entirely. Two groups of metal–ceramic heaters and temperature sensors (PT1000) are distributed within shell-1 and shell-2 for controlling the temperatures at the middle and bottom of the cartridge. Additionally, since the nozzle is at the furthest point from the heat source, heat loss at the nozzle is always the severest. This often leads to the solidification of thermoplastic polymers inside the nozzle, which interrupts the printing process. Hence, to preserve the heat around the nozzle area, a shell made of aluminum alloy (shell-3) was installed to cover the nozzle [25]. The proposed print head was installed on the three-axis linear stage of a self-developed high-precision 3D bioprinter (SIA bioprinter PRO, as demonstrated in Figure 1b), and pneumatic pressure was employed to actuate the printing process for the printing trial in this research. 

## 3. Thermal Analysis of the Print Head

### 3.1. FEM Modeling for the Print Head

**Figure 2:**
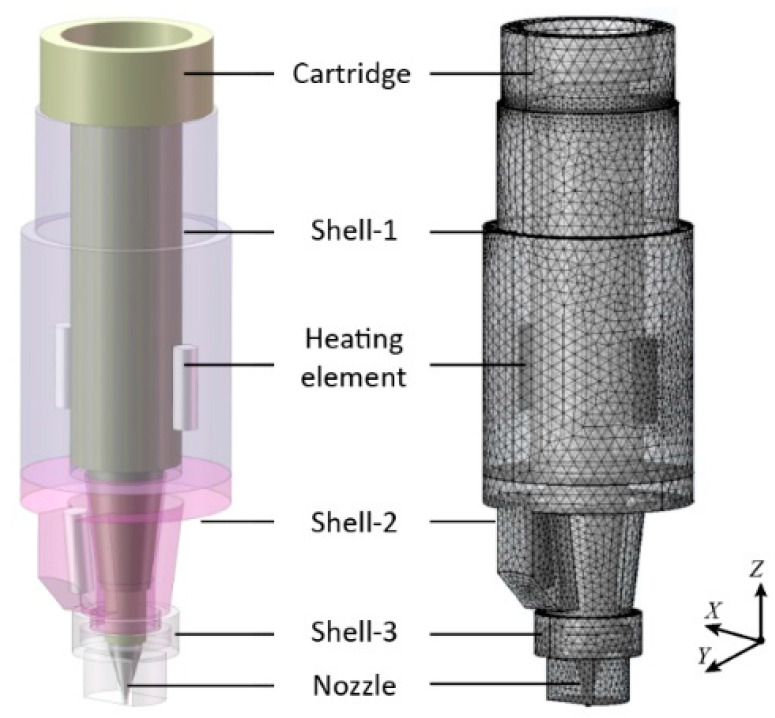
Simplified model and computational mesh of the print head.

To calculate the temperature distribution of the proposed print head, a FEM thermal simulation model was established based on the COMSOL Multiphysics 5.6 (COMSOL, Burlington, MA, USA). To facilitate the modeling process and reduce calculating time, the 3D digital models of the print head were simplified by SOLIDWORKS 2020 (Dassault Systèmes SolidWorks Corporation, Concord, MA, USA). Parameters of the material properties are listed in Table 1. Geometrical features which have little impact on the temperature distribution in the print head, such as screw holes and chamfers, were omitted to simplify the model of the print head, as demonstrated in Figure 2. The total number of nodes in the computational mesh of the proposed model, which is auto generated in the COMSOL, came to 2,022,000. In addition, the thermal boundary conditions of the simulation were investigated to improve the accuracy of the FEM model. The regions location near the heating elements were simplified as a heat boundary with a predefined temperature value. The interfaces between each component of the print head were considered thermal contact boundaries. Surfaces of the print head exposed to the atmosphere were regarded as convective heat transfer boundaries.

### 3.2. The Boundary Conditions of the FEM Model

#### 3.2.1. The Heat Source of the Print Device

Temperature sensors were installed near the heating elements to accurately control the temperature where the heating elements are located during the process of heating the bio-ink materials in the cartridge. This means the regions of the three heating elements in the FEM model can be approximately considered as heat sources with predefined temperature values. 

#### 3.2.2. Thermal Contact Conductance

The heat generated by the heating elements gradually transfers to the entire print head and raises the temperature of the bio-ink materials accommodated in the cartridge. The two components, which are in contact at a nominally flat surface, only touch each other at several discrete spots. Considering the surface condition of components, the widely adopted Cooper–Mikic–Yovanovich contact theory [27] was employed to define the thermal contact conductance:

(1) $$h_{c}=1.25k_{contact}\frac{m}{\sigma }{\left(\frac{p}{H_{c}}\right)}^{0.95}$$

where $h_{c}$ is the thermal interfacial contact conductance; $k_{contact}$ is a parameter that can be calculated with the heat conductivities of the basic materials via software; m is the number of microscopic contact regions; σ is a parameter to evaluate the profile roughness, which is related to the processing method; p is the pressure between the two solid bodies; and $H_{c}$ is the microhardness near the interface. Specifically, the roughness profile of mechanical parts was processed to Ra 1.6; the corresponding m and σ can be acquired from mechanical manuals. Further, the p at the horizontal interface can be estimated by calculating the ratio of the upper element weight and the interface area. Additionally, $H_{c}$ can be substituted with the Vickers hardness of the selected aluminum alloy. The defined values which were adopted for these parameters are listed in Table 2.

#### 3.2.3. Convection Heat Transfer Coefficients

In the 3D printing process for thermoplastic polymers, the temperature of the print head inevitably becomes higher than that of the surrounding air. This results in heat convection between the external surfaces of the print head and the ambient air. Two dimensionless numbers—the Reynolds number ($R_{e}$) and the Grashof number ($G_{r}$)—are calculated to determine the regime of the heat convection [28]. Since the estimated value of $G_{r}/R_{e}{}^{2}$ is much larger than 1, the type of heat convection is regarded as natural convection. For the vertical planes and cylinders in the FEM model, the convection heat transfer coefficient is calculated using the equation [29]:

(2) $$h_{v}=\frac{k}{L}{\left\{0.825+\frac{0.387{\left(G_{r}P_{r}\right)}^{1/4}}{{\left[1+{\left(0.492/P_{r}\right)}^{9/16}\right]}^{8/27}}\right\}}^{2},$$

where k is the heat conductivity of air; $P_{r}$ is the Prandtl number of the air related to the temperature, which can be searched for using software; and L is the characteristic length, which is utilized to express the geometric characteristics of the surface exposed to the air. Additionally, for the upper surface of the horizontal plates in the FEM model, the heat transfer coefficient can be defined as followed [30]:

(3) $$h_{p}=\frac{k}{L}0.54{\left(G_{r}P_{r}\right)}^{1/4}\;\;(10^{4}\leq G_{r}P_{r}\leq 10^{7}),$$

(4) $$h_{p}=\frac{k}{L}0.15{\left(G_{r}P_{r}\right)}^{1/3}\;(10^{7}\leq G_{r}P_{r}\leq 10^{11}),$$

and for the lower surface of horizontal plates in the model:

(5) $$h_{p}=\frac{k}{L}0.27{\left(G_{r}P_{r}\right)}^{1/4}\;(10^{5}\leq G_{r}P_{r}\leq 10^{10}).$$

**Figure 3:**
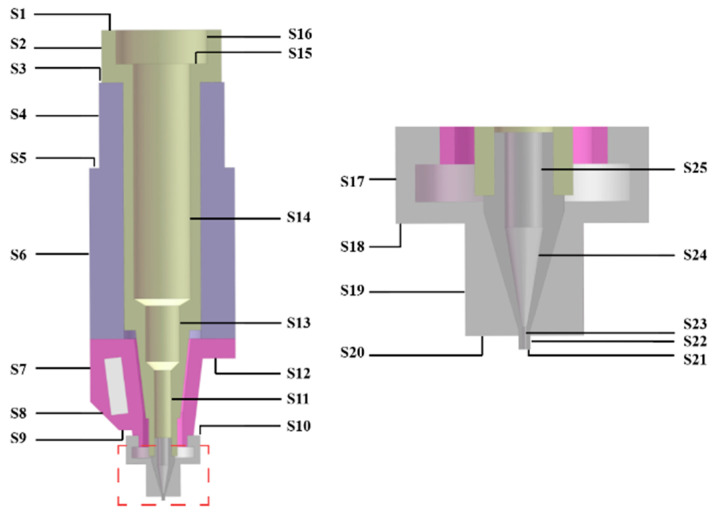
The numbering of external surfaces.

Additionally, since the angle of the inclined walls in the model range from 45° to 90°, all these inclined walls are regarded as vertical planes or cylinders. The location of these surfaces is indicated in Figure 3. The type of each external surface was determined, and the calculated L values are listed in Table 3. 

#### 3.2.4. Calculation Results

**Figure 4:**
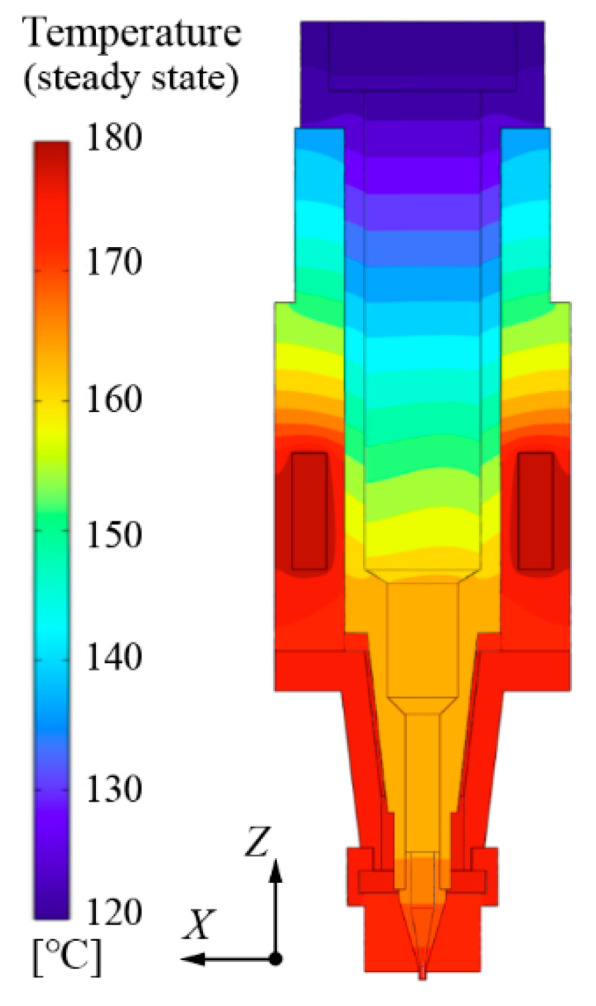
Temperature distribution with a heat source temperature of 180 °C.

Figure 4 exhibits the steady-state temperature distribution in the print head. A cross-section on the X–Z plane in the Cartesian coordinate system was selected in the FEM model to present the temperature fields of the print head. The temperature of two groups of heat sources were both preset to 180 °C. Because the chemical structure of the molecule chains of the thermoplastic polymers is prone to be destroyed under high temperature, the maximum value of the controlled temperature should be limited. Generally, the printing process for the thermoplastic polymers which are employed in the fabrication of biocompatible scaffolds can be conducted at a temperature below 180 °C [22,23,24], hence, the maximum heating temperature in the simulation was set to 180 °C. 

It can be observed that the temperature at the top of the cartridge was always the lowest, since the upper end of the cartridge is utilized to connect with the pipeline and exposed to the air. Although the temperature distribution near the top of the cartridge is non-uniform and cannot be controlled, it has little effect on the 3D printing process. In the thermoplastic polymer extrusion process, the material in the cartridge flows from the top to the bottom of the print head. This means the materials near the upper end of the cartridge will ultimately pass by the high-temperature area around the bottom of the print head. This indicates that the temperature fields around the heating elements are more significant to the extrusion process. In this area, the temperature at the nozzle is higher than that in the middle area of the cartridge, which means the temperature distribution in the print head presents poor uniformity. The deviation of temperature leads to a difference in the rheological properties of printing materials at varying locations, which is prone to affect the accuracy of the diameter of the printed filament. Therefore, the uniformity of temperature distribution in the proposed print head should be further improved.

## 4. A Novel Temperature Control Strategy Based on the Proposed Print Head 

### 4.1. A Novel Temperature Control Strategy

According to the configuration of the proposed print head, the preset temperature of two groups of heat sources can be controlled separately. This means that the temperature distributions around the heat sources can be adjusted separately. Consequently, the overall temperature field of the print head can be regulated. To achieve this, a temperature strategy is proposed to improve the temperature distribution uniformity in the proposed print head. 

As indicated in Figure 1a, the diameter of the cartridge decreases gradually from its middle to its bottom. This means that the bulk of the bio-ink materials are accommodated in the middle area of the cartridge. Therefore, the temperature of the heat source in shell-1 ($T_{I}$) has a dominant impact on the overall temperature distribution of the print head. Furthermore, since the two groups of heat sources are located close to each other, the interaction between them should also be taken into consideration when presetting $T_{I}$ and $T_{II}$ (the temperature of the heat source in shell-2). When the metal–ceramic heaters in shell-1 are the only activated heat sources, the area surrounding the metal–ceramic heater located in shell-2 is also heated to a certain temperature ($T_{II}^{\prime }$) due to heat conduction. According to the control logic of the heat source, once the temperature near the temperature sensor is higher than the predefined value, the heat source will not continue to generate heat. Therefore, when the heat source in shell-2 is activated, the preset $T_{II}$ must be higher than $T_{II}^{\prime }$. As can be seen in Figure 4, $T_{II}^{\prime }$ was about 164.75 °C when $T_{I}$ was preset to 180 °C. Hence, several values for $T_{II}$, ranging from 165 °C to 180 °C, were selected to investigate the temperature distribution uniformity under varying conditions, as shown in Table 4.

### 4.2. Simulation Results and Discussion

**Figure 5:**
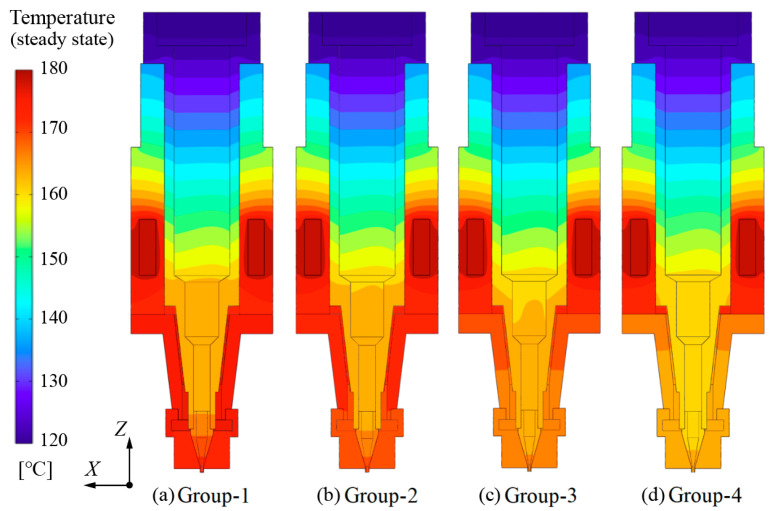
Temperature distribution of the print head in various groups.

The calculation results are shown in Figure 5. It can be seen from Figure 5a,b that the high-temperature area around the nozzle shrank when $T_{II}$ decreased from 180 °C to 175 °C. This means the gap in temperature between nozzle area and the middle of the cartridge was preliminarily eliminated. As shown in Figure 5c, with the further decline of $T_{II}$, the temperature distribution at the bottom of the cartridge became much more uniform. Ultimately, the temperature at the location around the cartridge bottom became almost consistent when $T_{II}$ was set at 165 °C in group-4. 

**Figure 6:**
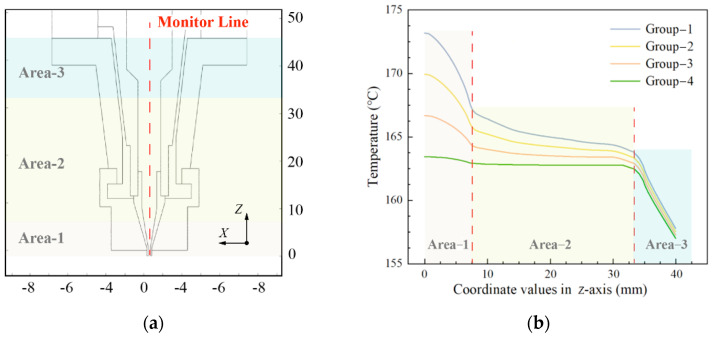
Quantitative comparison of temperature distribution in the cartridge: (**a**) schematic diagram around the bottom of the print head; (**b**) temperature curves of the monitor line.

To further present the feasibility of the proposed temperature control strategy for improving the temperature distribution uniformity, the temperature distributions under varying heating conditions are quantitatively compared in Figure 6. As shown in Figure 6a, three areas were marked based on the coordinate values in the z-axis, and a monitor line was located at the central line of the cartridge. The temperature curves of the monitor line are shown in Figure 6b. 

It can be seen that the temperature curves of different groups indicate a more obvious deviation in area-1 and area-2 with the variation of $T_{II}$, and the temperatures of area-1 show a higher value than that of other areas because the heat of shell-2 can be transferred to shell-3 via heat conduction. This indicates that the proposed temperature control strategy mainly affected the temperature distribution in area-1, and had limited impact on that of area-3. Furthermore, all the temperature curves declined sharply in area-1 except that of group-4. The slopes of the curves then decrease gradually with the increase of coordinate value on the *z*-axis in area-2. Finally, the temperature curve in each group declines with a similar slope when the monitor line reaches area-3. The above phenomena demonstrate that the temperature curve in group-4 manifests a stable and smooth trend around area-1 and area-2. This means the temperature uniformity at the bottom of the cartridge is significantly improved with the novel temperature control strategy. 

### 4.3. Experimental Verification for the Simulation Results

**Figure 7:**
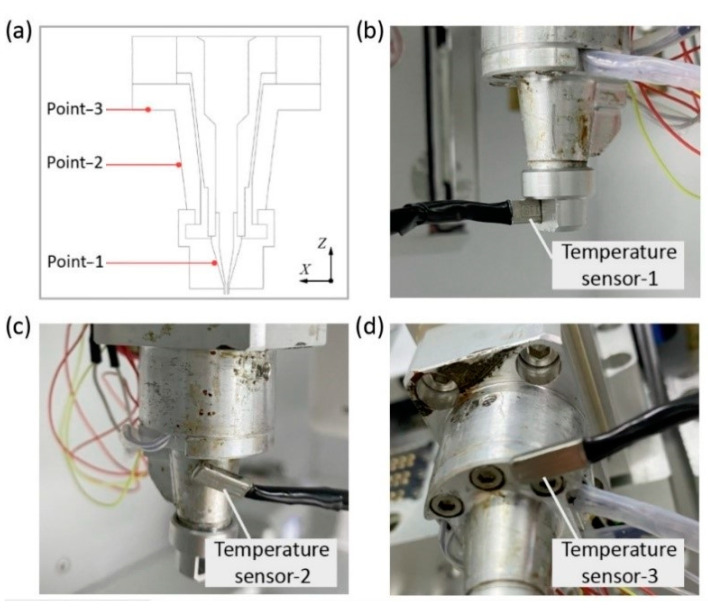
Monitor points and the experiment setup. (**a**) locations of the observing points; (**b**) temperature sensor for point-1; (**c**) temperature sensor for point-2; (**d**) temperature sensor for point-3.

The temperatures at three points in the print head were measured to verify the accuracy of the simulation results. On account of the small structural dimensions of the print head, it was impractical for temperature sensors to reach the internal surface of the cartridge. Hence, the temperature of the external surface of the print head was measured as a compromise. The locations of these three monitor points are marked in Figure 7a. Point 1 is located at the interface between the nozzle and shell-3. Point 2 and point 3 are located at the external surface of shell-2. As demonstrated in Figure 7b–d, three temperature sensors were used to carry out the temperature measuring experiment. The temperature sensors were located at the positions of the monitor points, as shown in Figure 7a. 

**Figure 8:**
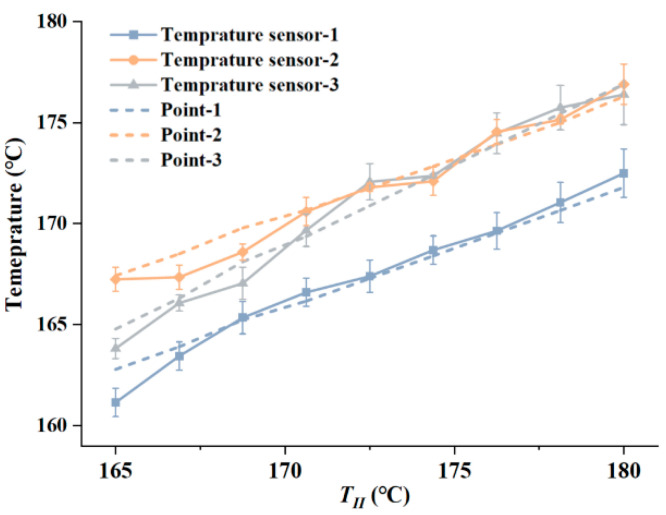
Temperature measurements and calculation results.

Figure 8 shows the measured temperature curves and the FEM simulation results. During the measuring process, $T_{I}$ was set to 180 °C and $T_{II}$ was set to 165 to 180 °C. For each test condition, temperature sensor data was recorded every ten seconds after the print head reached thermal balance. The recording time for each test condition was thirty minutes, and the average temperature during this period was regarded as the test result. The dashed lines represent the temperatures curves at the monitor points with varying $T_{II}$ in the FEM model. The solid lines indicate the tested temperature curves in the measuring experiment. Comparing the experimental curves and the calculation results, it can be seen that the experimental curves nearly overlap with that acquired by simulation, which verifies the reliability of the FEM calculation results. 

## 5. Printing Trial Based on the Proposed Print Head

As the rheological properties of thermoplastic polymers are sensitive to the spatial temperature distribution of print head, a non-uniform temperature field within the print head will definitely affect the process of forming a continuous filament. The proposed print head configuration and temperature control strategy aim to alleviate this phenomenon and improve the accuracy of printed filament diameters. To validate their feasibility, extrusion printing trails with and without the novel temperature control strategy were separately implemented, and the results are presented in this section. 

### 5.1. Rheological Investigation of PLCL

PLCL is a well-known type of thermoplastic polymer with excellent biocompatibility and degradation properties. It is often adopted in printing trials as a bio-ink material. To explain the printing phenomenon in printing trials, the rheological properties of PLCL were first investigated. As a blend of PCL and poly(L-lactide) (PLLA), 50:50 PLCL pellets (Daigang Biomaterial Co., Ltd., Jinan, China) were adopted due to the better melting efficiency of this formulation [31,32]. 

According to the simulation results shown in Figure 6b, the temperature curve for the print head without the novel temperature control strategy in group-1 demonstrated a substantial temperature difference in area-1 and area-2. This means the rheological properties of printing materials in the print head will differ greatly according to location. Hence, the mean values of temperatures in area-1 and area-2 for group-1 were calculated as temperature-1 and temperature-2, respectively. Meanwhile, for the printing process utilizing the novel temperature control strategy in group-4, the temperatures in area-1 and area-2 were similar in value. The mean temperature of these two areas was defined as Temperature-3. The three temperatures defined for rheological investigations are presented in Table 5. 

**Figure 9:**
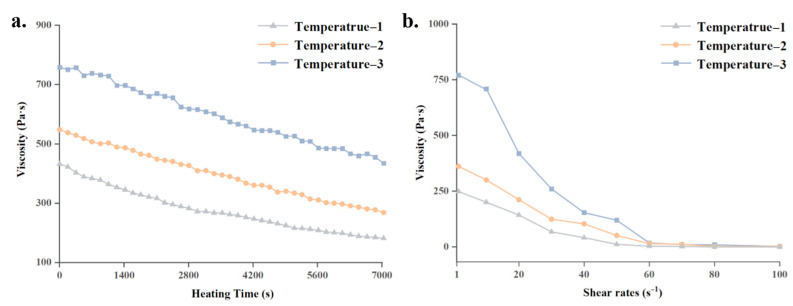
Rheological properties of PLCL: (**a**) viscosity curve of PLCL with varying heating time and temperature; (**b**) viscosity curve of PLCL with varying shear rate and temperature.

A rotation rheometer (HAAKE MARS III, Thermo Fisher Scientific, Shanghai, China) was employed to measure the viscosity of PLCL melts. The viscosity curves at the selected temperatures with varying heating times and various shear rates are presented in Figure 9. To acquire the viscosity of PLCL melts during the heating process, viscosity curves with increasing heating time and varying temperatures were measured via dynamic rheological testing at a steady shear rate of 1 s^−1^, and the observation time in rheology test was over 1.5 h, which is identical with the preheating period of printing trials. Figure 9a demonstrates obvious distinctions among the PLCL viscosity curves at various temperatures during the heating stage, with material in areas with a higher temperature showing a lower viscosity. The reason for this phenomenon is that the higher temperature of the heated PLCL molecules means they have more energy overcome the attractive forces between them, which leads to a decline in the viscosity of the melts. Additionally, the viscosity also declines with increasing heating time, which can be explained by the thixotropic character of PLCL melts. In a period of continual heating, the polymer conformation of each PLCL molecule disentangles and its mass center begins to move, which also leads to a decline in the material’s viscosity.

To further investigate the viscosity of PLCL melts during the printing process, steady shear sweeps from 1 to 100 s^−1^ at the chosen temperatures were conducted. The obtained viscosity curves with the variation of shear rates are presented in Figure 9b. It can be seen that the viscosity of the melt at each temperature decreased sharply at first with the growth of shear rate and subsequently became steady. Under the actuation of shear force, PLCL polymer chains disentangle and demonstrate shear thinning behavior, which causes a drop in viscosity. This phenomenon indicates that the viscosity of PLCL melts will show poor uniformity inside the cartridge due to the non-uniform temperature distribution.

### 5.2. The Result of Print Trial

Before the 3D printing trials, the PLCL pellets were inserted into and heated in the cartridge for over 1.5 h to ensure they were entirely melted. A nozzle with a diameter of 300 μm was adopted for the extrusion experiment. During each printing trial, the pneumatic pressure was set to 0.2 MPa, and the movement speed of the print head was preset to 3.3 mm/s. Pictures of the printed structures were taken with a digital camera and an optical microscope (Phenix, Shangrao, China). 

**Figure 10:**
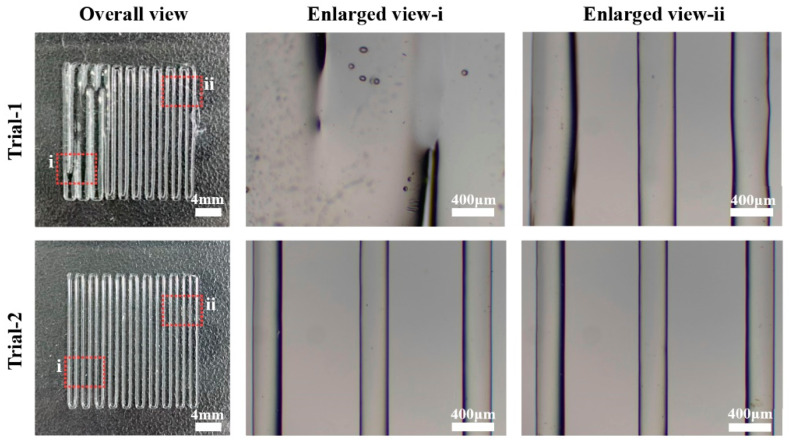
Photographs and micrographs of printed structures.

In order to verify the efficiency of the temperature control strategy, two groups of trials were conducted, with the $T_{I}$ and $T_{II}$ of group-1 and group-4 as listed in Table 4. As shown in Figure 10, the PLCL filaments were printed from left to right. Micrographs of the areas around both ends of the printed filament (enlarged view-i and view-ii) were taken to observe the print results of materials located at area-1 and area-2, respectively, as shown in Figure 10b.

Trial 1 was conducted with the preset temperatures in group-1. It can be seen that the sizes of filaments in the enlarged view-i are much larger than that in the enlarged view-ii, which shows a worse uniformity in the size of printed filaments. The widths of the extruded filaments in enlarged view-i are so large that these filaments are fused, and the diameter of both filaments in enlarged view-i and view-ii are not perfectly stable. Trial 2 was achieved with the temperatures in group-4, which is characterized by a high temperature distribution uniformity at the bottom of the print head. The micrographs of the printed filaments indicate excellent diameter accuracy and a smooth surface. 

The above phenomena can be explained as follows. As indicated in Figure 9, the viscosity of PLCL melts is sensitive to variation in temperature. For PLCL melts in the nozzle of the print head, a lower viscosity leads to a higher flow velocity when actuated under an unvarying pressure [33]. As a result, the print head extrudes a higher volume of melts out of the nozzle per unit time, which results in wider printed filaments. In trial-1, the rheological properties of PLCL in enlarged view-i and enlarged view-ii reflect the viscosity curves for temperature-1 and temperature-2 in Figure 9, respectively. Due to the lower viscosity of the PLCL melts in enlarged view-i, these printed filaments are of greater width than that in enlarged view-ii. In trial-2, the printed filaments shown in enlarged view-i and enlarged view-ii were printed at a similar temperature. The rheological properties of the material along the entire filament reflect the viscosity curves for temperature-3 in Figure 9, which means the velocity of PLCL flow during the printing process was relatively consistent. This led to the uniform diameter and shape of the printed filaments. This verifies that the proposed print head and temperature control strategy can improve the temperature distribution uniformity, which consequently improves the diameter uniformity of printed filaments.

## 6. Future Work

In this study, the uniformity of 3D printed structures made from a specific type of thermoplastic polymer was improved by means of a novel temperature control strategy. By controlling the temperature distribution in the print head, printed filaments with a consistent diameter and smooth surface were produced. In our future work, a wider range of bio-ink materials will be printed using the developed print head to further verify its efficiency. Furthermore, the mechanical properties of the printed filament will be investigated and improved by optimizing the print parameters, such as printing temperature and pneumatic supply pressure. Moreover, the printed architectures and scaffolds will be tested in order that their the biomechanical properties can be enhanced.

## 7. Conclusions

In this research, the spatial uniformity of temperature distribution in a 3D print head was investigated via thermal analysis based on an FEM simulation model. A novel print head construction was proposed, along with a temperature control strategy. The ability of these proposals to improve the uniformity printed filaments’ diameter was further validated in printing trails. The main conclusions of this paper are as follows:

(1) The temperature of the two groups of heat sources in the novel print head configuration developed for this study can be regulated separately.
(2) It was verified that the temperature control strategy, proposed on the basis of FEM simulation results and a temperature measuring experiment, can regulate the temperature distribution through the separate control of the two heat resources.
(3) When the temperature of heat sources at the middle and bottom of the print head were defined as 180 °C and 165 °C, respectively, the uniformity of temperature distribution was significantly improved.
(4) The printing trials for PLCL melts showed that higher uniformity of temperature distribution around the nozzle area can improve the uniformity of printed filament diameters.

## Figures and Tables

**Table 1:** Parameters selected for the simulation.

Material	Thermal Conductivity	Density	Thermal Capacity
Aluminum alloy	201 W/(m·K)	2700 kg/m^3^	900 J/(kg·K)

**Table 2:** Defined values of parameters.

Parameters	Values
σ	1.6 μm
m	0.4
p	100 kPa
$H_{c}$	107 HV

**Table 3:** Calculated *L* values of each surface.

Surface	Type	L/mm
S1	Horizontal plate	1.25
S2	Vertical cylinder	10.50
S3	Horizontal plate	1.14
S4	Vertical cylinder	9.00
S5	Horizontal plate	8.04
S6	Vertical cylinder	50.00
S7	Vertical cylinder	20.00
S8	Vertical plane	1.50
S9	Horizontal plate	0.43
S10	Horizontal plate	0.75
S11	Vertical cylinder	4.90
S12	Horizontal plate	3.09
S13	Vertical cylinder	4.90
S14	Vertical cylinder	79.24
S15	Horizontal plate	0.75
S16	Vertical cylinder	1.73
S17	Vertical cylinder	3.20
S18	Horizontal plate	1.09
S19	Vertical plane	5.70
S20	Horizontal plate	1.80
S21	Horizontal plate	0.08
S22	Vertical cylinder	0.40
S23	Vertical cylinder	1.01
S24	Vertical cylinder	4.75
S25	Vertical cylinder	4.70

**Table 4:** Defined temperatures in the FEM model.

Group	$T_{I}$	$T_{II}$
1	180 °C	180 °C
2		175 °C
3		170 °C
4		165 °C

**Table 5:** Defined temperatures for rheological investigations.

Group	Value
Temperature-1	170.0 °C
Temperature-2	166.3 °C
Temperature-3	162.8 °C

## Data Availability

Not applicable.
